# Progress in Medicine

**Published:** 1901-09-07

**Authors:** 


### Progress in Medicine.

(*Continued from page* 363.)

**Intermittent lameness of vascular origin** was first described by Charcot in 1854. It is a rare condition and but few cases have been described since. Putnam[3] has recently recorded an additional instance. The subjects are usually past middle life, who after walking for five to ten minutes are seized with a cramp-like feeling in the legs which is so severe as to render further progress impossible. After a few minutes' rest they can walk again. Examination of the legs usually reveals signs of defective arterial supply, thus one of the pedal arteries may be pulseless and the foot cool and slightly pale, or red or blue. The occurrence of the symptoms during exertion may be due to the need of the muscles for an increased supply of blood or to the induction of vascular spasm. The disorder is chronic. Improvement may sometimes be produced by measures which tend to promote the circulation—iodide of potassium, warmth, galvanism with the feet resting in warm water, and tonics. In Putnam's case these methods produced no amelioration, which was brought about by massage, so that ultimately he could walk one or two miles without difficulty.

**Effect of Severe Athletic Exercise in the Young.** In the course of a discussion on this subject Pembrey[4] said that the dyspnœa of exertion was not due to an accumulation of carbonic acid gas in the lung but was of cardiac origin. "Second wind" was entirely a question of compensation between the heart and the respiratory pump. An increase of blood pressure and of temperature occurred during exercise. Broadbent said that the first effects of dyspnœa arose from the too rapid forcing of blood into the right side of the heart from pressure on the veins by the muscles, the respiratory pump being unable to effect its removal sufficiently rapidly. The second wind came when the right heart had unloaded itself and redistribution of blood had taken place, this being helped by relaxation of the arterioles. In everyone there existed a line beyond which exercise was injurious, and that line was indicated by continuation of the dyspnœa and loss of sleep. Damage to the heart might easily be done by severe exercise, especially when taken by persons when they were in bad health.

**Malignant Endocarditis.**—Jackson has summarised the clinical and pathological features of some 59 cases of malignant endocarditis observed in the Boston City Hospital. In about half the cases a necropsy showed the presence of predisposing chronic heart disease. In the early stages of many cases the symptoms appeared to have suggested pulmonary tuberculosis, cough being a frequent symptom often accompanied by bloody expectoration. Vomiting and severe sweating, either with or without rigors were frequently present and delirium was common. Special diagnostic value should be paid to the examination of the blood, leucocytosis being invariably present, whilst it is absent, or at least very rare, in typhoid fever and acute tuberculosis. In the differential diagnosis of the disease the most important points are: (1) Evidence of some heart lesion in the past, especially if there has recently been some infective or suppurative disease: (2) such early symptoms as fever, rigors, joint pains, vomiting, cough and weakness; (3) in the later stages irregular fever, rigors and cough, with delirium, stupor and petechiæ; (4) leucocytosis.

[3] Putnam, Boston Med. and Surg. Jour., Feb. 21, 1901. [4] Pembrey, Brit. Med. Jour. [5] Jackson, Med. and Surg. Reports of the Boston City Hospital, 11th series.

